# Potential of adipose-derived stem cells in muscular regenerative therapies

**DOI:** 10.3389/fnagi.2015.00123

**Published:** 2015-07-13

**Authors:** Sonia-V Forcales

**Affiliations:** Genetics and Epigenetics of Cancer, Institute of Predictive and Personalized Medicine of CancerBarcelona, Spain

**Keywords:** adipose, stem cells, muscle, regeneration, reprogramming, transdifferentiation, transplantation

## Abstract

Regenerative capacity of skeletal muscles resides in satellite cells, a self-renewing population of muscle cells. Several studies are investigating epigenetic mechanisms that control myogenic proliferation and differentiation to find new approaches that could boost regeneration of endogenous myogenic progenitor populations. In recent years, a lot of effort has been applied to purify, expand and manipulate adult stem cells from muscle tissue. However, this population of endogenous myogenic progenitors in adults is limited and their access is difficult and invasive. Therefore, other sources of stem cells with potential to regenerate muscles need to be examined. An excellent candidate could be a population of adult stromal cells within fat characterized by mesenchymal properties, which have been termed adipose-derived stem cells (ASCs). These progenitor adult stem cells have been successfully differentiated *in vitro* to osteogenic, chondrogenic, neurogenic and myogenic lineages. Autologous ASCs are multipotent and can be harvested with low morbidity; thus, they hold promise for a range of therapeutic applications. This review will summarize the use of ASCs in muscle regenerative approaches.

## Introduction

One major challenge of modern medicine is to repair damaged tissues. Self-adult stem cells for regenerative therapies could overcome transplantation limitations such as organ rejection or shortage of organs available for donation. Furthermore, many degenerative diseases such as muscular dystrophies have no cure and the possibility to replace damaged muscle of the whole body by transplantation, nowadays, is not feasible. In dystrophic patients, continuous myogenic regeneration is sustained by resident stem cells. However, stem cell pool is eventually depleted, which correlates with aggravation of the disease and deterioration of patients. Consequently, cellular regenerative therapies that may replenish myogenic stem cell populations and counteract muscle loss by producing healthy muscle could represent a new treatment to improve quality of life and prevent early death for dystrophic patients. The potential use of adult adipose-derived stem cells (ASCs) for muscle regeneration will be discussed.

## Myogenic Progenitors from Different Sources

Intensive research has been done to identify different types of myogenic progenitors in muscles that could be isolated, manipulated and expanded *in vitro* for therapeutic use reviewed in (Motohashi and Asakura, 2014). The classical stem cell from muscle is the satellite cell (Mauro, 1961; Montarras et al., 2005; Scharner and Zammit, 2011), however, in recent years several groups have described other myoprogenitors in muscle tissue such as muscle side-population cells (Gussoni et al., 1999; Asakura and Rudnicki, 2002), muscle-derived stem cells (Qu-Petersen et al., 2002), interstitial cells (Mitchell et al., 2010), and muscle-derived CD133 + stem cells (Benchaouir et al., 2007). Other cells present in muscles called fibroadipogenic progenitors have been shown to crosstalk with satellite cells to enhance myogenesis (Joe et al., 2010; Uezumi et al., 2010). Although muscle tissue seems to contain a high concentration of progenitors (it is estimated that 550 satellite cells are present in 1 mg of muscle tissue, Bentzinger et al., 2012), muscle biopsies are difficult to obtain and are small, therefore the yield of progenitors that can be obtained seems to be insufficient for regenerative therapies. One approach to solve this hurdle would be to expand progenitor population’s *ex-vivo*, but unfortunately it has been shown to result in low engraftment rates and failure to improve muscle strength in Duchenne muscular dystrophy (DMD) patients (Mendell et al., 1995; Vilquin, 2005). Interestingly, growing those progenitors in different extracellular matrices and hydrogel platforms that better mimic *in vivo* niche, could overcome this problem (Gilbert et al., 2010). Nevertheless, satellite cells from Duchenne dystrophic patients proliferate less, undergo rapid senescence (Cossu and Mavilio, 2000) and are difficult to obtain due to fiber fragility (Boldrin and Morgan, 2012). In addition, muscle biopsies are still painful and can cause irreversible damage to donors.

Therefore, additional sources of myogenic stem cells have been explored such as embryonic stem cells (ESCs) and induced pluripotent stem cells (iPS). Ectopic expression of two regulators of myogenic transcription, BAF60c and MyoD, were able to differentiate hESC towards myogenic lineage, resulting in three-dimensional contractile structures called myospheres (Albini et al., 2013). Whether those myospheres could engraft in damaged muscle and contribute to regeneration was not tested, nevertheless they could be a tool for drug discovery to identify molecules that potentiate myogenesis. In addition, myospheres generated from dystrophic patients’ iPS cells could open the way for tailored treatments in the future. Overexpression of paired-box transcription factors such as PAX3 (master regulator of embryonic myogenic program) in mouse ESCs, and PAX7 (involved in maintenance of satellite cell compartment) in human ESC and iPS cells, converted these cells to myogenic lineage. Those cells were then transplanted into immunodefficient Duchenne muscular dystrophic mice, engrafted successfully to supply dystrophin and repaired muscle strength. Importantly, in case of PAX7-ESCs/-iPS the stem cell pool was restored (Darabi et al., 2008, 2011, 2012). However, safety use of these genetically modified embryonic or pluripotent cells needs to be carefully addressed before they can be used in clinical setting. Some concerns using these former approaches are related to lentiviral usage for genetic modification of cells and the need to fully reprogram them in order to avoid teratoma formation and tumor progression when transplanted to patients.

A safer and ethical-acceptable alternative could be the use of adult multipotent stem cells such as mesenchymal stem cells (MSCs), which are self-renewable and still able to differentiate to several lineages *in vitro*. MSCs were first identified in bone marrow (BM) by Friedenstein and colleagues (Friedenstein et al., 1966, 1968), and subsequently MSCs have also been found in many other adult tissues such as adipose, synovial membrane, dermis, periosteum, dental pulp, peripheral and menstrual blood, liver, spleen and lung (da Silva Meirelles et al., 2006; Kassis et al., 2006; Zou et al., 2010). One of most extensively studied MSC is the BM-derived, which has been shown to differentiate towards myogenic lineage *in vitro* (Pittenger et al., 1999). BM transplants contributed to muscle regeneration in cardiotoxin-injured muscles and in *mdx* mice (a model for DMD; Ferrari et al., 1998; Bittner et al., 1999; Gussoni et al., 1999; Fukada et al., 2002; Bossolasco et al., 2004). However, engraftment efficiency was low and further research estimated that muscle repair by BM-derived cells did not exceed 1% of total muscle fibers during the lifespan of transplanted *mdx* mice. In addition, this procedure did not contribute to ameliorate muscular dystrophy symptoms (Ferrari et al., 2001). Most of these works used whole BM populations and not purified MSCs; thus, it is possible that limited myogenic potential was due to little MSCs presence in total BM cells, being less than 0.01% in harvests that approximately yield 6 × 10^6^ cells per mL (Pittenger et al., 1999). In order to increase myogenic engraftment of transplanted cells, BM-derived MSCs were expanded and genetically modified *ex-vivo* to express PAX3. When transplanted in dystrophic mice, PAX3 BM-derived MSCs were able to activate myogenic program and to fuse successfully with injured muscles, showing higher engraftments (10.8 + 3.6%) than previous works. Surprisingly and unfortunately, the expected functional recovery was not observed (Gang et al., 2009).

Another MSC type is adipose-derived mesenchymal stem cell, which is now termed ASC. ASCs were characterized by Patricia Zuk and colleagues, who isolated ASCs from lipoaspirates, expanded them *in vitro* and showed their multipotentiality towards not only adipogenic but also osteogenic, chondrogenic, neurogenic and myogenic lineages upon culture with defined media (Zuk et al., 2001). Could ASC represent a new and powerful source of myogenic progenitors for regenerative therapies?

## Adipose Stem Cell Characterization

Compared to harvesting BM-derived MSCs, which requires drilling into the bone, ASCs can be obtained by liposuction, which is a less invasive procedure and may be more attractive for donors due to its positive esthetic results. In addition, stem cell yields are higher from adipose tissue, with 1 g containing an average of 2 × 10^6^ cells with 10% being ASCs (Aust et al., 2004; Oedayrajsingh-Varma et al., 2006). For this reason, adipose tissue represents an abundant and practical source of multipotent stem cells for autologous and heterologous cell transplantation approaches compared to other tissues.

**Figure 1:**
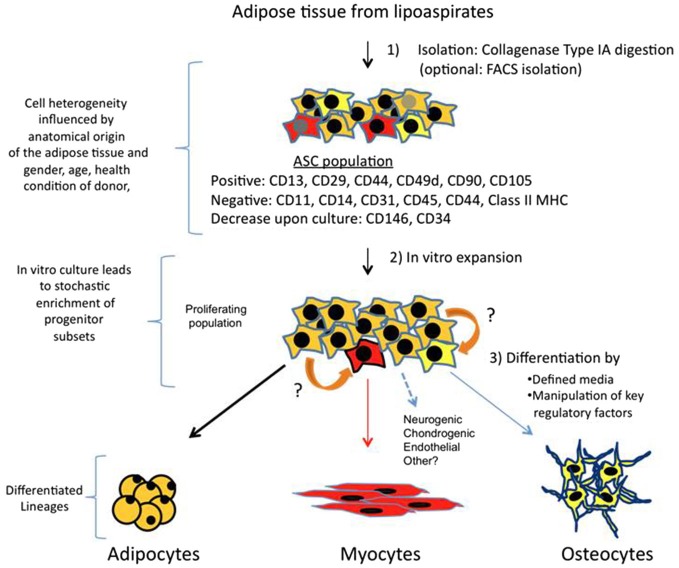
**Isolation, expansion and differentiation of ASCs**. Adipose tissue is obtained from lipoaspirates but it can also be obtained from other surgeries. Upon digestion with collagenase type IA followed by centrifugation; the pellet obtained is known as SVF. SVF is a mixture of several types of progenitors and more differentiated cells. A majority of cells (90–100%) are positive for mesenchymal stem cell surface markers, and this fraction is the ASCs. Other markers such as CD146 and CD34 are more controversial, and may represent subsets of other progenitors, which vary in their ratios depending on anatomical origin of fat and other parameters related to donors. *In vitro* culture of this proliferating population, which can arrive to 70 PDs, can also alter percentages of specific progenitors. Therefore, differential expression of CD markers and their fluctuations may represent a heterogenic composition of ASCs, which contain subsets of multipotent cells that can respond to differentiation cues of other lineages (represented in red and yellow). Adipocyte is the main lineage obtained from ASCs, however, upon culture with defined media, or by ectopically expressing specific factors, myogenic, osteogenic and other lineages can also be obtained. It is also possible that progenitors can transdifferentiate (indicated by orange arrows) in response to lineage specific cues.

The protocol for ASCs isolation comprises an enzymatic digestion of adipose tissue or lipoaspirate, with collagenase type IA, followed by centrifugation (Figure 1). The pellet is called stromal vascular fraction (SVF), which contains a heterogeneous population of cells: fibroblast, red blood cells, smooth muscle cells, pericytes, and preadipocytes. Freshly isolated SVF can be plated with growth media (typically DMEM + 10% FBS) where a population of adherent cells proliferates and can be expanded for several passage doublings (PDs) in most cases without karyotype abnormalities (more than 70 PDs reported by; Rodriguez et al., 2005). This adherent population is considered to be ASCs, which is characterized to be positive for a specific set of surface mesenchymal markers: CD13, CD29, CD44, CD49d, CD90, CD105 and negative for hematopoietic markers such as CD11, CD14, CD31, CD45 and CD144 (Zuk et al., 2002; Katz et al., 2005; Mitchell et al., 2006; Yoshimura et al., 2006; Varma et al., 2007; Zannettino et al., 2008). Other CD markers have been more controversial and with expressions showing variable percentages amongst total ASCs population. For instance, many works report that CD146 is not expressed by ASCs, however there is data suggesting that a subset of ASCs express CD146 and localize to areas surrounding blood vessels (Crisan et al., 2008; Zannettino et al., 2008; Cai et al., 2011), arguing in favor that a pericyte multipotent population resides in adipose tissue. A CD34 + CD146- population with multipotent abilities has been detected as well in the outer adventitial ring of vasculature (Traktuev et al., 2008; Zimmerlin et al., 2010). Therefore, all these works suggest that several lineage precursors may be forming part of ASCs population (Zeve et al., 2009) and that their relative composition present in isolated SVF may reflect different anatomic origins of adipose tissues, as well as other characteristics associated to donors such as age, body mass index, gender and health conditions. In other words, the particular niche of adipose tissue has an important role to determine type and quantities of progenitors, and a more detailed analysis needs to be performed in order to fully understand which subtypes of progenitors are better suited for a specific regenerative aim.

Furthermore, it is important to note that upon culture for several weeks some CD markers have been reported to increase (CD105, CD166) while others such as CD146 and CD34 decrease dramatically (Mitchell et al., 2006; Varma et al., 2007). This can be explained by enrichment of certain cell types during culture where diverse parameters such as composition of basal media, supplements, plating substrates or cell confluence could have an impact on this issue. The media used for amplifying proliferating ASCs is a regular 10% FBS in DMEM, and using other media with specific growth factors and cytokines has not been tested systematically to understand how it could impact on maintaining or selecting a certain progenitor population. Conveniently, longterm cultured ASCs express very little amounts of major histocompatibility complex class I proteins and do not express class II proteins (HLA-DP, DM and DR), which are crucial for antigen presentation. As a result, absence of these molecules could give an advantage for ASCs therapeutic usage, allowing evasion of host’s immune response in heterologous transplantations (Lin et al., 2012). Whether ASCs would upregulate human leukocyte antigen (HLA) proteins upon myogenic differentiation is not known.

## *In vitro* Skeletal Muscle Differentiation Potential of ASCs

The ability of ASCs to differentiate *in vitro* towards myogenic lineage has been reported by several groups. Culture of ASCs with myogenic differentiation media has resulted in ASCs adopting an elongated morphology, similar to differentiating myoblasts, and expression of early (MyoD1, myogenin) and late (myosin heavy chain) markers of muscle differentiation (Zuk et al., 2001; Mizuno et al., 2002; Zheng et al., 2006). These and other works show variable differentiation efficiencies in terms of onset and fusogenic ability, which may be the result of using different inductive media (Table 1). Co-culture experiments with myoblasts have shown that secreted factors and cell-to-cell contacts induce ASCs myogenic conversion and fusion with mouse C2C12 and primary myoblasts (Lee and Kemp, 2006; Di Rocco et al., 2006; Eom et al., 2011). Variations in fusogenic ability were observed when hASCs were treated with myogenic differentiation media before co-culture, and the highest efficiency was obtained when ASCs were already at late stages of myogenic differentiation and expressing myosin heavy chain (MyHC) and dystrophin (Eom et al., 2011).

**Table 1:** **Summary of ASC’s myogenic differentiation, engraftment and functional assessment**.

	Differentiation, engraftment, functional assessment	Myogenic differentiation media
***In vitro***
Zuk et al. (2001)	12% myogenic differentiaton of hASCs from lipoaspirates.	DMEM, 10% FBS, 5% horse serum, 0.1 μM dexamethasone 50 μM hydrocortisone, 1% Antibiotic-antimycotic
Mizuno et al. (2002)	At 6 weeks of differentiation, aprox. 15% of hASCs expressed MyoD and 8.5% expressed Myosin heavy chain.	Same as Zuk et al. (2001) without dexamethasone.
Zheng et al. (2006)	After 6 weeks of differentiation, mouse ASCs expressed desmin monitored by immunofluorescence.	Same as Zuk et al. (2001).
Eom et al. (2011)	C2C12-GFP/hASC cocultures; at day 21, myotubes showed 50% nuclei from hASC origin.	LG-DMEM, 10% FBS, 3 μM 5-azacytidine (Sigma), 10 ng/ml FGF-2 (Sigma) for 24 h. Followed by 10% FBS, 10 ng/ml FGF-2 in LG-DMEM. Finally, hASC 5 days-filtered supernatant (conditioned media) was used.
Di Rocco et al. (2006)	Primary myoblasts and mASC-GFP direct cocultures showed 10-fold higher myotube incorporation of ASCs than transwell cocultures. Green myotubes expressed Troponin T by immunofluorescence.	DMEM high glucose, 5% horse serum, 2 mM L- glutamine, 1% penicillin-streptomycin.
Lee and Kemp (2006)	Positive fusion of hASCs with C2C12 myoblasts as myotubes expressed nestin from human origin monitored by immunofluorescence.	DMEM-high glucose (Gibco), 2% heat-inactivated horse serum (Gibco), and 1% penicillin/streptomycin.
Vieira et al. (2008a)	Coculture of DMD-derived myoblasts with GFP-hASCs. Fusion successful monitored by dystrophin expression in green myotubes by immunofluorescence.	hASCs passage 4 were cultured DMEM-HG, 10% FBS, 0.1 μM dexamethasone (Sigma), 50 μM cortisol (Sigma) and 5% HS (Gibco) for 45 days.
***In vivo***
Rodriguez et al. (2005)	hASC injected in tibialis anterior muscles from mdx mice. Dystrophin was detected in up to 50% of the myofibers analyzed per section 10 d after transplantation. 10 to 50 d post- transplantation, 73 to 85% increase in peripheral nuclei from hASCs; 27 to 15% decrease in central nuclei.	Skeletal Muscle Cell Differentiation medium (PromoCell).
Di Rocco et al. (2006)	mASC from 6-week-old GFP+ mice were injected, after hind limb ischemia, in the left adductor muscle of GFP negative mice. After one week, GFP positive fibers represented up to 20% of the total area of sections (38.33±8.82 GFP-positive fibers per mm^2^ of section area calculated as an average from 8 experiments). Dystrophin was detected in up to 10% of the myofibers analysed on sections (11.7±2.94% dystrophin positive fibers per mm2 of section area, average from 6 experiments).	DMEM high glucose, 5% horse serum, 2 mM L-glutamine, 1% penicillin-streptomycin).
Zheng et al. (2006)	mASCs-LACZ injected in muscles of mdx mice. Few cells engrafted.	Same as Zuk et al. (2001).
Liu et al. (2007)	Cardiotoxin-injured mdx muscles injected locally with Flk1+ ASC: dystrophin expression restored in many fibers. Central nuclei decreased compared to controls at 4 weeks post-transplantation: 48.1% vs. 72.8% (n=5), *p* = 0.05, and at 12 weeks post-transplantation: 43.3% vs. 74.2%, *n* = 5, *p* = 0.05. Decreased creatinine kinase concentrations in Flk1+ ASCs-treated mice compared to controls at 12 weeks post transplantation: 5,321.6 +/− 1,289.75 vs. 13,746.8 +/− 5,373.75 Ul^−1^, *n* = 5, *p* = 0.005).DMEM containing 2% fetal bovine serum (FBS), 5% horse serum (Gibco), 50μ M hydrocortisone (Sigma), 100 U/ml penicillin, and 100 μg/ml streptomycin (Gibco).
Vieira et al. (2008b)	Systemic delivery of hASC in SJL mice (tail vein injection). Human Dystrophin found in approx. 50% +/− 2% (*p* = 3.623 × 10^−13^, Student’s t test, *n* = 7) of the fibers.The treated group showed an improvement of 15.2% +/− 7.0% in their performance, whereas the untreated worsened 6.12% +/− 6.0% (*p* = 0.013, Student’s t test, *n* = 7).	Same as in Vieira et al. (2008a), during 10 days before transplantation to SJL mice. At this stage, hASCs cells express MyoD.
Goudenege et al. (2009)	Tibialis anterior muscles of Rag2^−/−^γC^−/−^ mice injected with MyoD-hASCs show between 27 and 38 spectrin-positive fibers per section whereas ASCs injected muscles show less than 10 spectrin-positive fibers per section.	1.5 g/l glucose Dulbecco’s modified Eagle’s medium supplemented with 10 μg/ml insulin and 5 μg/ml transferrin when the cells reached 90% confluence.
Vieira et al. (2012)	Systemic injections of hASC in GRMD dogs showed variable dystrophin expression by WB (11 to 19% where 100% corresponds to normal human muscle). Immunofluorescence for dystrophin showed compatible results. hASC local injection in muscles of GRMD dogs did not engraft (hASCs not present in muscles 1 month after transplantation).	Same as Zuk et al. (2001).

*Composition of myogenic differentiation media used in different works is also indicated*.

Importantly, for regenerative purposes as a treatment for dystrophies, ASCs should also be able to fuse with dystrophic myotubes, for this reason, Vieira and colleagues showed that *in vitro* human ASCs could indeed fuse with DMD-derived myoblasts to form myotubes that recovered dystrophin expression (Vieira et al., 2008a). Therefore, these experiments support the use of allogeneic ASCs not only for their conversion towards the myogenic lineage and thus contributing to counteract muscle loss, but also as providers of WT dystrophin and other potential beneficial factors that may be missing in dystrophic muscles. The use of coatings and hydrogels has shown to enhance myogenic differentiation from ASCs (Choi et al., 2012). However, at present, a universal media composition for a robust myogenic differentiation of ASCs is missing.

Table 1 summarizes the results showed in these works and indicates the composition of differentiation media used in each of them.

## *In vivo* Skeletal Muscle Differentiation Potential of ASCs

All these *in vitro* works indicate that different stimuli can potentiate and promote ASCs differentiation towards myogenic lineage, ranging from hormones and growth factors present in media (or secreted by cells), to cell-to-cell contacts or even by other mechanophysical inputs such as plating surfaces. However, whether ASCs could contribute to myogenic regeneration *in vivo* was not clear until the work of Rodriguez et al. (2005). In this work, the authors characterized *in vitro* human ASCs from different young donors and prior to transplantation in *mdx* mice, they identified two multipotent populations, one characterized to be fast adherent and expandable *in vitro* for more than 200 passages (chosen for transplantation experiments), and a slower adherent population that showed senescent features upon long term culture. Interestingly, fast-adherent ASCs at low-passages were HLA-I positive and HLA-II negative whereas at later passages were HLA-I low and HLA-II negative. Therefore, to assess whether absence of major histocompatibility complex could be advantageous to evade host immune’s system, the transplantations were performed in *mdx* mice that were treated or not with 10 mg of cyclosporine A/kg (daily i.p. injection) to immunosupress the host. Human ASCs (1.5 × 10^5^) from long-term passages (160 PDs) were injected in left tibialis anterior (TA) muscle. Transplanted animals showed expression of dystrophin at several days post-transplantation, and human nuclei (elegantly revealed by FISH with a specific probe for human centromeres) were observed in central and peripheral locations of muscles, indicating that human-derived ASCs contributed to the regenerative process. After 6 months, dystrophin expression was well distributed along muscle fibers, and even present in adjacent gastrocnemius muscle, suggesting that cell migration from injection site to other dystrophic muscles could have occurred or that dystrophin delivery was achieved by another unknown mechanism. Furthermore, in immunocompetent mice, regeneration took place successfully with no evidence of CD3 positive-lymphocytic infiltration. In contrast, hASC from early passages (that express HLA-I antigens) elicited an immune response in the host and could not restore dystrophin expression. Whether hASCs contributed to regeneration not only by fusion but also by converting themselves to myogenic lineage *in vivo* was not clear, although *in vitro* conversion of ASCs to myogenic lineage suggests that both mechanisms (fusion to myotubes and also myogenic conversion of ASC themselves) could have occurred. In this regard, the work of Liu et al. (2007), suggests that not only fusion but also myogenic conversion of hASC occurs *in vivo* since expression of myogenic markers (MyoD, Myogenin, MyHC) from human origin could be detected by RT-PCR with specific primers. In this work, a subset of hASCs expressing endothelial marker Flk1 were transplanted by two different methods in two different regeneration models: tail vein injection (systemic) in cardiotoxin-injured TA muscles or intramuscular injection (local) in *mdx* mice. They both resulted in successful engraftment, partial recovery of sarcolemmal expression of dystrophin, decreased necrosis after 4 weeks and lower levels of muscle creatinine kinase in blood after 12 post-transplantation weeks. Importantly, the satellite stem cell pool seemed to be replenished from hASC since positive PAX7 cells co-stained for the human protein β2M. Another work (Vieira et al., 2008b), also transplanted hASCs by tail-vein injection in a SJL mice, a model of limb girdle muscular dystrophy 2B which is milder than Duchenne’s. Different from *in vitro* co-culture experiments where highest fusion was observed using hASCs differentiated to late myogenic stages (Eom et al., 2011), undifferentiated hASC, but not hASC previously differentiated to myogenesis *in vitro*, were able to engraft successfully in muscles and provide expression of dysferlin and dystrophin of human origin. This data suggests that a progenitor state is more suitable to use in transplantation approaches, perhaps this undifferentiated population is more competent to receive regenerative signals from *in vivo* niche than more differentiated cells do, and this “instructive” step may be necessary in order to fuse to injured muscle. Also, it is possible that myogenic-differentiated ASCs are not able to sustain a proliferating pool of cells that could give cycles of regeneration upon time, which may be required to achieve robust and successful engraftments. These authors also reported that inflammatory process shown by those dystrophic muscles from SJL mice, did not improve upon hASC transplantation, in contrast to other works, which have reported a beneficial immunoprotective effect of ASC transplantation, for instance in rat brains upon a hemorrhagic stroke (Kim et al., 2007) and in an osteoarthritic mice model (ter Huurne et al., 2012). Nevertheless, mice showed improved muscle functionality in several motor ability tests up to 6 months of transplantation.

To assess the viability of cell transplantation approaches in humans, it is crucial to validate whether they can be feasible in larger animal models. This issue is important in order to predict pathogenesis and treatment outcomes. An alternative bigger model than mice and rats for muscle regeneration is the golden retriever muscular dystrophy (GRMD) dog. This model reproduces full spectrum of human DMD and it has been used for successful transplantation studies with systemically injected mesoangioblasts, which is a type of mesenchymal stem cell present in walls of large vessels. In these experiments, dystrophin expression was recovered as well as partial muscle functionality (Sampaolesi et al., 2006). However, the dogs were immunosuppressed and treated with steroids, which have been shown a beneficial impact on muscle functionality. For this reason, Vieira et al., 2012, assayed the transplantation of hASCs in GRMD dogs that were not immunosuppressed. hASCs were injected systemically into the dog cephalic vein (5 × 10^7^ cells/kg in 0.1 ml of Hank’s buffered salt solution) and engrafted successfully in the muscles. Expression of human dystrophin was validated up to 6 months after transplantation but could not be found after 12 months, suggesting that hASCs were not able to replenish the stem cell pool. The percentage of human dystrophin expressed in muscles was small (ranging from 6% to 19%) as monitored by immunofluorescence and immunoblotting. Unfortunately, histopathological features were not improved and beneficial effects on disease symptoms were difficult to be concluded. Therefore, from these data one can speculate that perhaps multiple injections, for instance every 6 months, and larger amounts of cells may be required to maintain exogenous dystrophin expression at levels that give a positive functional output. Surviving animals did not present adverse immune responses or other complications after 24 months, which might indicate ASC transplantation to be a safe procedure. In contrast, locally injected hASC were not able to engraft successfully, opposite to what had been described for *mdx* mice (Rodriguez et al., 2005; Liu et al., 2007).

Table 1 summarizes the results showed in these works and indicates quantifications of ASCs contribution to muscle differentiation and regeneration.

## MyoD-Driven Conversion of ASCs

From all these works it is suggested that ASCs can be reprogrammed to myogenic lineage *in vitro* and *in vivo* by extracellular cues, highlighting a possible therapeutic use of ASCs as a new source of myogenic progenitors. However, efficiency of this conversion is not 100%, suggesting that there is a subpopulation of ASCs receptive to myogenic-commitment cues and a non-responsive population. In order to increase levels of myogenic conversion, Goudenege et al., 2009, genetically manipulated ASCs to express MyoD1, a transcriptional factor crucial for myogenic differentiation. MyoD1-pioneering ability was suggested years ago by Weintraub and colleagues, who ectopically expressed MyoD1 in fibroblasts, fat, liver and nerve cell lines resulting in activation of myogenic gene expression (Weintraub et al., 1989, 1991). Fibroblasts harboring an inducible MyoD1 system and μdystrophin gene were able to convert to myogenic lineage and contribute to muscle regeneration in *mdx* mice (Kimura et al., 2008). In ASCs, overexpression of MyoD1 resulted in upregulation of CD56, a surface marker of myogenic progenitors committed to differentiate (Capkovic et al., 2008), from 2% to 30% of total ASCs (Goudenege et al., 2009). Those MyoD1-expressing hASCs could efficiently form multinucleated myotubes *in vitro* upon culture with differentiation media (containing 10 μg/ml insulin and 5 μg/ml transferrin when cells reached 90% confluence), as well as to fuse with human DMD-derived myoblasts *in vitro*. Upon transplantation in a mouse model for regeneration (cryoinjury in TA muscles of Rag2^−/−^γC^−/−^ immunodeficient mice), hASC engraftment was monitored by immunofluorescence against human Lamin A/C, revealing that hASC localized between fibers and also integrated in regenerating muscles. To assess hASC contribution to regenerative process, several human specific muscle proteins (spectrin, dystrophin, and δ-sarcoglycan) were detected by immunofluorescence at the membrane of different fibers. Muscles transplanted with MyoD1-hASCs seemed to contain higher number of positive human-derived myogenic marker-expressing fibers than WT-hASCs, indicating that MyoD1 expression in ASCs results in higher regenerative *in vivo* potential. This data argues in favor of manipulating ASCs *in vitro* with transcriptional factors to force ASCs commitment towards myogenic lineage, since using these manipulated ASCs result in a higher contribution to muscle regeneration. Whether those MyoD1-ASCs contribute to functional recovery of damaged muscles remains to be tested and perhaps MyoD1-hASC should also be assayed in DMD models such as *mdx* mice and GRMD dogs.

## Epigenetics of ASC-Derived Myocytes

During adult stem cell differentiation as well as in the progression from a pluripotent to a multipotent state, expression of proliferative and pluripotent genes is erased, and expression of differentiation or lineage-specific genes from previously silent loci is activated. This process is controlled by concerted action of signaling cues with ubiquitous and cell-specific transcription factors that govern a changing epigenetic landscape during cellular differentiation. In other words, distinct epigenetic signatures can be associated to a precise cellular stage (Hussein et al., 2014; Tee and Reinberg, 2014).

DNA methylation at CpG sites is one of best-studied epigenetic modifications, which has been associated to gene repression (Heard et al., 1997; Walsh et al., 1998; Gaudet et al., 2003), although it is also found in gene bodies of highly transcribed genes (Jjingo et al., 2012). DNA methylation fluctuates during early mammalian development (Reik, 2007), but it is quite stable in most of the cells, which show an average of 70–80% of methylated CpGs. Nevertheless, global analyses in different cell types and tissues have shown that a dynamic regulation occurs for almost 22% of autosomal CpGs at enhancers of lineage specific regulators (Hon et al., 2013; Ziller et al., 2013; Carrió et al., 2015), suggesting that DNA methylation at CpG sites is an epigenetic feature that determines cellular identity.

Therefore, to verify a certain lineage reprograming, conversion, or proper differentiation, instead of monitoring expression of a few markers of these processes, global characterization of gene expression and epigenetic patterns seems to be a more complete approach to validate whether a full or partial conversion has been achieved. Berdasco and colleagues pursued this technique and characterized global DNA methylation status of myogenic lineages derived from hASC by Infinium methylation arrays interrogating 27,578 CpG sites (Berdasco et al., 2012). Their results indicate that methylome of ASCs-derived myocytes is more similar to the one of undifferentiated ASCs than to the one of primary myocytes from human biopsies. ASCs methylome shared the lowest similarity with a rhabdomyosarcoma cell line, a pediatric muscle tumor. Importantly, methylomes of ASCs and ASCs-myogenic derivatives did not show hypermethylation of tumor suppressor genes, a hallmark of DNA methylation profiles of cancer cells. Hence, these results argue in favor of ASCs as a safe cell population for therapeutic purposes. Intriguingly, these data show that although ASC-derived myocytes and primary myocytes present overlapping methylomes, ASC-derived myocyte methylome is more similar to the one of undifferentiated ASCs. Therefore, this result could imply several interpretations such as incomplete conversion towards myogenic lineage of all ASCs, or that just a subset of isolated ASCs was converted to myogenic lineage while the rest were resistant to this conversion. It could also imply that many of the observed 2,313 differential methylated regions between ASC-derived myocytes and primary myocytes, are not essential for myogenic differentiation, which indeed took place in ASC-derived myocytes supported by positive expression of α-sarcomeric actin, actinin and troponin by immunofluorescence. In any case, other epigenetic mechanisms different from DNA methylation may be crucial for lineage plasticity contributing to reprogram ASCs towards myogenic lineage. In fact, a direct link between DNA methylation state and differentiation capacity is not obvious as for instance mesenchymal stem cells from adipose, BM and skeletal muscle all show lineage-specific genes hypomethylated (Sørensen et al., 2010).

A comparative epigenomic profiling in murine and human models of adipogenesis, which monitored genome-wide distribution of several histone modifications, CTCF and adipose specific transcriptional factor peroxisome proliferator-activated receptors (PPAR_γ_), was able to identify two novel players, promyelocytic leukaemia zinc finger (PLZF) and serum response factor (SRF), with anti-adipogenic activity (Mikkelsen et al., 2010). A positive correlation was found between gene expression values and presence of H3K27ac in enhancer regions, giving to this modification a predictive value when present. This work also suggested that gain of histone acetylation accompanying PPAR_γ_ binding, distinguishes functional PPAR_γ_ binding sites from nonproductive ones. Surprisingly, a major number of PPAR_γ_ binding sites were different in mouse and human, and many were associated to rodent-specific transposable elements. This work shows that adipogenesis may not be 100% equivalent in mice and humans, implying that regulation of ASC plasticity may be different as well. Whether PLZF and SRF could play a role in early stages of myogenic determination of ASCs remains to be elucidated.

## Considerations for Use of ASCs in Muscle Regenerative Therapies

Previously mentioned studies support the idea that ASCs are a promise for muscle regenerative therapies: they can differentiate towards myogenic lineage, they can be obtained in great quantities by simple liposuction, and they may represent a safer alternative to embryonic or induced pluripotent stem cells, which can produce tumors if full reprograming is not achieved. Autologous transplantations of ASCs have been safely used for reconstructive surgery and wound healing therapies for several years (Ross et al., 2014), nevertheless, clinical studies are scarce to confirm long-term safeness of ASC transplants (Barkholt et al., 2013). Whether allogeneic ASC transplants in humans would work for muscular dystrophy treatment is not known, but successful engraftments of ASCs in muscles of immunocompetent mice support the idea that they might.

However, for successful muscular regenerative therapies it is necessary that transplanted ASCs not only engraft in muscles but also that differentiate efficiently resulting in a positive functional output. At present, ASCs’ efficiency to differentiate towards myogenic lineage is low and their contribution to recover muscle function in *in vivo* regenerative models is not consistent. Stem cell research from skeletal muscle and hematopoietic fields have reported that stem cells are heterogeneous regarding self-renewal, proliferation and lineage output (Copley et al., 2012; Malecova and Puri, 2012), supporting a view of mixture of progenitors in those tissues. Adipose tissue is emerging as a new crucial player in the endocrine field, and the former idea that it is just a reservoir of energy, in form of lipid deposits, is changing. Therefore, it is not surprising to find different types of progenitors also present in this complex tissue. ASCs comprise a mix of multipotent progenitors, which seem to vary in their composition depending on several factors such as donor characteristics or their anatomical origin. Perhaps, anatomical location defines subsets of stem cells, which may respond better to signals of that particular environment. Supporting this idea, ASCs derived from epicardial adipose tissue differentiate poorly towards adipocyte lineage, whereas they express myocardial and endothelial markers *in vitro*. Furthermore, when transplanted into injured hearts they are able to differentiate into cardiac cells, engraft successfully and promote paracrine effects to induce local vascularization in mouse, rat and porcine models of myocardial infarction (Bayes-Genis et al., 2010, 2013). Consequently, not all adipose tissue from the body has the same characteristics and more research is needed to identify molecular determinants that define their specificities. It is quite plausible that extracellular niche plays an important role in this issue; however signaling pathways that control stem cell pools in adipose tissue as well as their crosstalks with adjacent normal or diseased organs are still poorly understood. In regenerating dystrophic muscles of *mdx* mice, recently identified fibroadipogenic progenitors play a crucial role by enhancing muscle progenitor differentiation as well as modulating the inflammatory response that leads to fibrosis (Mozzetta et al., 2013). Also, parabiotic experiments suggest that age-related changes in systemic environment and the niche in which progenitor cells reside prevent complete activation of these cells for successful muscle regeneration (Conboy et al., 2005). Thus, these and other works show that tissue homeostasis involves interplay of local and systemic factors, and it is far more complex than previously anticipated.

*In vivo* skeletal muscle regenerative experiments using ASCs suggest that there is a subset of cells with increased myogenic capacity, which argues in favor of a myoprogenitor population present in ASCs. Therefore, identifying this myoprogenitor ASCs with specific surface markers could allow their isolation and expansion for a successful therapeutic use. Encouragingly, for bone regenerative purposes, Chung and colleagues have identified a population of CD90-high expressing ASCs, which show increased osteogenic capacity in mice models of bone regeneration (Chung et al., 2013). However, regarding ASC-derived myoprogenitors it is not clear which marker may identify them. Myoprogenitor marker CD56 is only present in 2% of cultured ASCs (Goudenege et al., 2009), which seems a small percentage of cells to account for the observed ASCs myogenic capacity. Intriguingly, in ASCs there is a 29% of CD146 + cells (Zannettino et al., 2008), but whether those cells contain type II pericytes with ability to regenerate muscles after injury (Birbrair et al., 2013) is not clear. Flk1 + cells purified from ASCs showed high myogenic differentiation potential, and although Flk1 is considered to be an endothelial marker (Yamaguchi et al., 1993), cardiac stem cells seem to express Flk1 (Iida et al., 2005). Perhaps, single cell analyses similar to those performed for muscle stem cells (Sacco et al., 2008) could help to identify and characterize a group of myoprogenitor markers in ASCs that could be instrumental to isolate a progenitor fraction with enhanced myogenic properties more suitable for muscle regenerative goals.

Other works suggest that transdifferentiation of ASCs towards myogenic lineage is also possible, although whether this transdifferentiation includes reprograming to a myoprogenitor or direct differentiation is not clear. While transcriptional programs that direct adipocyte and myocyte differentiation are well studied and many players have been identified such as insulin-like growth factor 1 (IGF-1), bone morphogenetic protein (BMP), transforming growth factor beta (TGFB), retinoic acid, integrins, Ncadherin, galectin, cAMP response element-binding protein (CREB), PPARg, p38 kinase, MyoD, BAF60c, myocyte enhancer factor-2 (Mef2), ASH2 (Rosen and MacDougald, 2006; Perdiguero et al., 2009; Cristancho and Lazar, 2011; Dilworth and Blais, 2011; Giordani and Puri, 2013), early molecular events that determine which fate ASCs will follow are mostly unknown. Shed new light on this issue and showed that inhibition of Rho-ROK signaling pathway in differentiating myocyte precursors (C2C12 in presence of IGF1), blocked myogenic differentiation in favor of adipogenesis, while excessive Rho activation in adipocyte precursors (L1–3T3) blocked adipogenesis Sordella et al. (2003). The phosphorylation status of p190-B RhoGAP would be a sensor of this pathway. Other works have shown that in a mesenchymal stem cell population, a dominant negative RhoA expression induces adipogenesis whereas a constitutively active RhoA drives osteogenic differentiation (McBeath et al., 2004). These works highlight important roles of Rho pathway modulating cell fate decisions in MSC. Whether this pathway controls adipose vs. myogenic fate of ASC in response to IGF1 remains to be elucidated.

One of the signaling pathways involved in cell fate during development and also in cancer disease is Sonic Hedgehog. Eric Olson’s group has described that constitutive activation of Sonic Hedgehog signaling in adipose lineage results in skeletal muscle tumors that resemble embryonal rhabdomyosarcomas (Hatley et al., 2012). This work supports a transdifferentiation hypothesis of an adipose progenitor towards muscle lineage, and importantly it reminds us the need to make sure ASC progenitors used for therapy are not harboring mutations or activated signals that can lead to cancer. *In vitro* and *in vivo* assays show that ASCs are not intrinsically tumorigenic cells, however they could enhance tumorigenic behavior of c-Met+ breast cancer cells by eliciting an inflammatory microenvironment that sustained tumor growth (Eterno et al., 2014). In other words, using ASC in esthetic procedures for breast reconstruction after cancer could enhance recurrence at least in c-Met+ tumors. Thus, more research is needed to better understand ASC biology upon transplantation and ensure safeness for clinical applications.

Epigenetic modifications are catalyzed by enzymes that can be targeted by drugs; hence, these drugs have an impact on cell fate. For instance, use of demethylating drug 5-Azacytidine in fibroblasts resulted in their conversion to chondrocytes, adipocytes and contractile muscle striated cells (Taylor and Jones, 1979) illustrating the broad effect this drug had on demethylation of many lineage regulatory loci. Therefore, in order to control a specific lineage conversion at will, it is necessary to find more precise strategies. In this regard, CRISPR-CAS technology holds great potential for targeted approaches to gene therapy. Perhaps it could be used for several objectives ranging from recruiting desired epigenetic enzymes to specific lineage regulatory loci to activate known myogenic regulators in ASCs, or to target dystrophin gene in order to fix its mutations in autologous DMD-cultured ASCs. Alternatively, this strategy could also be used to activate expression of myogenic booster genes (Engvall and Wewer, 2003) in ASCs, which could then fuse to muscles to alleviate muscular dystrophy symptoms.

In conclusion, several works have shown promising data on ASCs contribution to muscle regeneration. ASCs safety is supported by *in vivo* transplantation experiments and epigenetic data. Identification of new protocols and tools that allow enrichment, expansion and manipulation of ASC-derived myoprogenitors could help to boost muscle regeneration.

## Conflict of Interest Statement

The author declares that the research was conducted in the absence of any commercial or financial relationships that could be construed as a potential conflict of interest.
